# Sex-Specific Cardiometabolic Profiles and Severity of Liver Fibrosis

**DOI:** 10.1001/jamanetworkopen.2026.0863

**Published:** 2026-03-09

**Authors:** Somaya Albhaisi, Steve Kim, Norah Terrault, Jennifer L. Dodge

**Affiliations:** 1 Division of Gastrointestinal and Liver Diseases, Department of Medicine, Keck School of Medicine, University of Southern California, Los Angeles; 2 Department of Population and Public Health Sciences, Keck School of Medicine, University of Southern California, Los Angeles

## Abstract

**Importance:**

Advanced liver fibrosis is increasing globally, with women experiencing faster progression despite lower prevalence. Cardiometabolic risk factors (CMRFs) may be differentially associated with fibrosis risk by sex.

**Objective:**

To examine sex differences in the association between individual CMRFs and significant liver fibrosis among US adults.

**Design, Setting, and Participants:**

This population-based, cross-sectional study was conducted using data from the US National Health and Nutrition Examination Survey, 2017 to 2020. Adults aged 20 years or older with valid transient elastography measurements were included. Data were analyzed from July 2024 through December 2025.

**Exposures:**

CMRFs, including high waist circumference (>102 cm in men or 88 cm in women), glucose intolerance, hypertension, hypertriglyceridemia, and low high-density lipoprotein cholesterol levels, were assessed, as well as obesity (body mass index ≥30 or ≥27.5 for Asian participants) and the presence of 2 or more CMRFs.

**Main Outcomes and Measures:**

Clinically significant fibrosis was defined as a liver stiffness of 8.0 kPa or greater by transient elastography. Multivariable logistic regression evaluated associations between CMRFs and significant fibrosis, adjusting for age, sex, race and ethnicity, smoking, and alcohol and testing CMRF by sex interactions.

**Results:**

The study population of 5981 participants included 2992 women (weighted percentage: 50.2% [95% CI, 48.2%-52.2%]; 14.7% Hispanic [95% CI, 12.0%-18.0%], 11.0% non-Hispanic Black [95% CI, 8.2%-14.7%], and 65.0% non-Hispanic White [95% CI, 59.4%-70.3%]; mean age, 49 years [95% CI, 48-50 years]) and 2989 men (weighted percentage: 49.8% [95% CI, 47.8%-51.8%]; 16.4% Hispanic [95% CI, 13.2%-20.2%], 9.4% non-Hispanic Black [95% CI, 7.3%-11.9%], and 64.6% non-Hispanic White [95% CI, 59.6%-69.3%]; mean age, 47 years [95% CI, 46-48 years]). Women had higher prevalence of high waist circumference (69.0% [95% CI, 66.1%-71.7%] vs 48.6% [95% CI, 44.3%-53.1%]) and lower prevalence of hypertension (41.0% [95% CI, 38.2%-43.8%] vs 44.9% [95% CI, 41.5%-48.4%]), glucose intolerance (31.1% [95% CI, 28.7%-33.5%] vs 41.7% [95% CI, 38.6%-44.9%]), and hypertriglyceridemia (36.6% [95% CI, 34.0%-39.3%] vs 48.8% [95% CI, 44.6%-52.9%]) compared with men. The prevalence of significant fibrosis was 6.9% (95% CI, 5.4%-8.8%) in women and 10.7% (95% CI, 8.8%-12.9%) in men. The point estimates in the association with significant fibrosis were significantly greater in women vs men for high waist circumference (adjusted odds ratio [aOR], 13.45 [95% CI, 5.70-31.78] vs aOR, 4.44 [95% CI, 3.00-6.57]; *P* for interaction = .01), glucose intolerance (aOR, 2.94 [95% CI, 1.64-5.28] vs aOR, 1.51 [95% CI, 1.08-2.13]; *P* for interaction = .045), and the presence of 2 or more CMRFs (aOR, 10.22 [95% CI, 4.76-21.95] vs aOR, 2.87 [95% CI, 1.91-4.31]; *P* for interaction = .002).

**Conclusions and Relevance:**

In this study, the presence of 2 or more CMRFs was associated with a greater increase in risk of significant fibrosis for women compared with men. Central adiposity and glucose intolerance were also associated with greater increases in risk among women.. These findings support the need for sex-specific screening approaches.

## Key Points

**Question:**

Are there sex-specific differences in associations between cardiometabolic risk factors and significant liver fibrosis among US adults?

**Findings:**

In this cross-sectional study of 5981 US adults from the 2017 to 2020 National Health and Nutrition Examination Survey, women had higher odds of clinically significant fibrosis in association with central adiposity, glucose intolerance, and the presence of multiple cardiometabolic risk factors compared with men.

**Meaning:**

These findings suggest that women with multiple cardiometabolic risk factors may face a disproportionately higher risk of liver fibrosis, supporting the need for sex-specific screening and prevention strategies.

## Teaser

This cross-sectional study investigates differences between men and women in the association of cardiometabolic risk factors with significant liver fibrosis.

## Introduction

The increasing incidence of advanced liver fibrosis and cirrhosis represents a significant public health challenge, with mortality rates from these conditions increasing globally. Notably, recent epidemiological data from 2018 to 2021^1,2,3^ reveal a concerning trend; while men have a higher overall prevalence of liver disease, rates of cirrhosis and its complications, such as hepatocellular carcinoma, are increasing more rapidly in women. This disparity suggests that underlying risk factors may be differentially associated with liver health in men and women, yet a lack of awareness of these differences may contribute to under-recognition and missed opportunities for early intervention.

A primary driver of this growing burden of liver disease is the increasing prevalence of cardiometabolic risk factors (CMRFs), which include overweight or obesity, elevated fasting glucose levels, high blood pressure, hypertriglyceridemia, and low high-density lipoprotein (HDL) cholesterol levels. The accumulation of these factors is associated with an increase in odds of the development of metabolic dysfunction–associated steatotic liver disease (MASLD) and progression of hepatic fibrosis in the general population.^4,5^ While all 5 CMRFs are associated with liver injury, emerging data suggest that they do not contribute equally to disease progression.^6,7^ Some factors, notably type 2 diabetes and visceral adiposity, have been associated with greater increases in risk of advanced liver fibrosis and adverse clinical outcomes.^5^ Type 2 diabetes has consistently been associated with greatest increases in risk of advanced fibrosis and liver-related mortality, with data from population-based cohorts showing a nearly 3-fold increase in the risk of clinically significant fibrosis among individuals with diabetes, independent of other metabolic traits.^8,9^ Similarly, visceral adiposity, rather than generalized obesity, has been implicated as a key driver of hepatic fibrogenesis due to its proinflammatory and insulin-resistant metabolic profile.^10^

Biological differences between men and women may explain the accelerated progression to advanced fibrosis observed in women,^11^ but the extent to which individual CMRFs may be differentially associated with liver fibrosis severity in men vs women remains largely unexplored. Addressing this knowledge gap is essential for improving risk stratification and guiding early, sex-specific intervention strategies to curb the rising tide of advanced liver disease.

In this study, we aimed to examine sex differences in the association between individual CMRFs and significant liver fibrosis among US adults using nationally representative data. By elucidating how these common risk factors may differentially contribute to liver fibrosis in men and women, our findings may inform more precise, sex-specific strategies for risk stratification and prevention in the general population.

## Methods

### Data Source and Study Population

This cross-sectional study used data from the National Health and Nutrition Examination Survey (NHANES), an ongoing, multistage probability survey capturing the civilian, noninstitutionalized US population. The analysis covered data collected for the 2017 to March 2020 NHANES cycles, during which unweighted response rates were 51% for the interviewed sample and 47% for the examination sample.^12,13^ Adults aged 20 years and older with valid transient elastography measurements were included in the study. Individuals were excluded if they had hepatitis B or C or incomplete data on CMRFs or alcohol use. We chose the 2017 to 2020 NHANES cycles because they included transient elastography measurements. We included only complete exams meeting standard reliability criteria as defined by NHANES: a fasting time of at least 3 hours, 10 or more valid stiffness measures, and a liver stiffness IQR/median stiffness <30%.^14^ This study did not constitute human participant research as determined by the University of Southern California Human Research Protection Program given that the study used secondary, deidentified, publicly accessible data and so was exempted from review and consent. This study is reported in accordance with the Strengthening the Reporting of Observational Studies in Epidemiology (STROBE) reporting guideline.

### Exposure Variables and Covariates

CMRFs were defined according to criteria from the Adult Treatment Panel III of the National Cholesterol Education Program.^15^ These included abdominal obesity, defined as high waist circumference (WC) of greater than 102 cm in men or 88 cm in women; hypertriglyceridemia, defined as triglyceride levels of 150 mg/dL or greater or use of lipid-lowering therapies; low HDL cholesterol levels, defined as less than 40 mg/dL in men or less than 50 mg/dL in women or use of lipid-lowering therapies; hypertension, defined as elevated blood pressure (≥130/85 mm Hg) or use of antihypertensive medications; glucose intolerance, defined as fasting glucose levels of 100 mg/dL or greater or use of diabetes medications; and the presence of 2 or more of these CMRFs (vs ≤1). Obesity (body mass index [BMI; calculated as weight in kilograms divided by height in meters squared] ≥30 or ≥27.5 for Asian participants) and high body roundness index (BRI; >4.0 for men and >5.0 for women) were also assessed but not included in calculating the number of CMRFs. We applied BRI thresholds of greater than 4.0 for men and greater than 5.0 for women, cutoffs that have been proposed in prior studies as optimal for identifying increased risk of MASLD.^16^ We set the threshold at 2 or more CMRFs to define early but clinically relevant metabolic dysregulation because major guidelines recommend fibrosis risk assessment in individuals with 2 or more metabolic risk factors (eg, obesity plus another factor) and because population-based studies show a dose-dependent increase in the prevalence and severity of liver fibrosis when moving from 1 to 2 or more CMRFs.^17,18,19,20,21,22^ Alcohol use was characterized as *above moderate* (exceeding the threshold for MASLD) if it was greater than 20 g/d for women and greater than 30 g/d for men and *excessive* if it was greater than 50 g/d for women and greater than 60 g/d for men.^23,24,25^ MASLD was defined as a controlled attenuation parameter of 288 dB/m or greater^26^ in the presence of at least 1 CMRF per the American Association for the Study of Liver Diseases/European Association for the Study of the Liver (AASLD/EASL) guidelines and alcohol use levels of 20 g/d or less for women and 30 g/d or less for men.^17,20,26^ Sociodemographic and health behavior data, including age, biological sex, race and ethnicity (Hispanic, non-Hispanic Black, non-Hispanic White, and other [defined as non-Hispanic other races, including Asian and multiple races]), smoking (defined as ≥100 cigarettes lifetime), alcohol use, poverty (defined as a ratio of family income to poverty value <1), and treatment for CMRFs, were collected during structured in-home interviews. Laboratory and anthropometric measurements were obtained during clinical examinations. In sensitivity analyses, we defined severe steatosis as a controlled attenuation parameter of 280 dB/m or greater.^27,28^

### Outcomes

The primary outcome was clinically significant liver fibrosis, defined as a liver stiffness measurement of 8.0 kPa or greater, a threshold previously validated for detecting significant fibrosis (stage ≥F2).^29,30^ This liver stiffness measurement cutoff aligns with current clinical guidelines and validation studies in populations with MASLD and alcohol-associated liver disease and EASL clinical practice guidelines to rule out advanced fibrosis.^31^

### Statistical Analysis

Survey statistical methods with variance estimated via Taylor-series linearization were used for all analyses to appropriately reflect the multistage, cluster-stratified sampling design of NHANES. Sex-stratified weighted means and percentages were estimated with corresponding 95% CIs for demographic features and CMRFs.

To explore sex-specific associations of CMRFs with fibrosis, the weighted prevalence of significant fibrosis was estimated stratified by sex and each individual CMRF and compared using the Pearson χ^2^ test with second-order Rao and Scott correction for the survey design. Age-standardized prevalence of significant fibrosis was also estimated by sex and CMRF using the direct method. Logistic regression models with CMRF by sex interactions were used to estimate sex-specific adjusted odds ratios (aORs) for the association of each CMRF with significant fibrosis while adjusting for a priori covariates of age, race and ethnicity, smoking, and alcohol use; these selected covariates were included based on their well-established associations with liver fibrosis risk, as demonstrated in large epidemiologic studies and clinical guidelines on liver disease.^4,32,33^ Models included sex interaction terms with each covariate to generate sex-specific estimates from a single pooled model. Multicollinearity was assessed via variance inflation factors (all <2). To account for potential confounding by socioeconomic status, we conducted a secondary multivariable analysis adjusting for poverty among the subset of participants with available data. We performed 4 sensitivity analyses to test the robustness of study results, with analyses among adults with severe steatosis (controlled attenuation parameter ≥280 dB/m), excluding participants with excessive alcohol use (>50 g/d for women and >60 g/d for men),^23^ among adults with MASLD, and exploring the BRI.^34^ Analyses were conducted using SAS statistical software version 9.4 (SAS Institute) and Stata/MP statistical software version 17.0 (StataCorp), with *P* < .05 from 2-sided tests considered statistically significant. Data were analyzed from July 2024 through December 2025.

## Results

**Figure 1.**
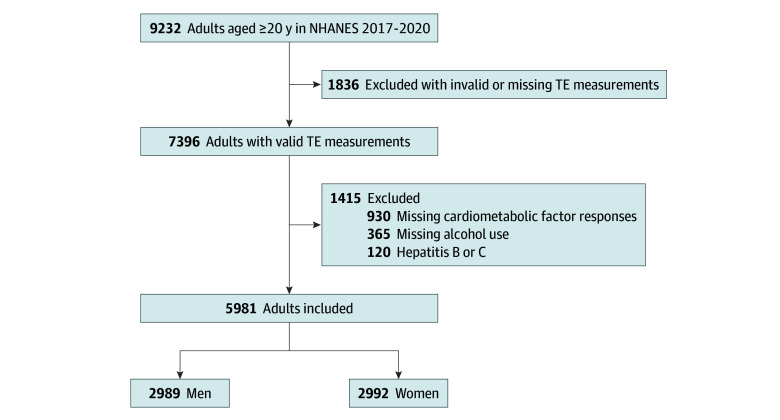
Study Flowchart NHANES indicates National Health and Nutrition Examination Survey; TE, transient elastography.

From the NHANES 2017 to 2020 survey years, an unweighted sample of 5981 adults was included in our analysis, consisting of 2992 women (weighted percentage: 50.2% [95% CI, 48.2%-52.2%]; 14.7% Hispanic [95% CI, 12.0%-18.0%], 11.0% non-Hispanic Black [95% CI, 8.2%-14.7%], and 65.0% non-Hispanic White [95% CI, 59.4%-70.3%]; mean age, 49 years [95% CI, 48-50 years]) and 2989 men (weighted percentage: 49.8% [95% CI, 47.8%-51.8%]; 16.4% Hispanic [95% CI, 13.2%-20.2%], 9.4% non-Hispanic Black [95% CI, 7.3%-11.9%], and 64.6% non-Hispanic White [95% CI, 59.6%-69.3%]; mean age, 47 years [95% CI, 46-48 years]). The selection of the analytic population is depicted in Figure 1. A total of 1295 participants excluded for missing responses to alcohol use or cardiometabolic data were similar in age and poverty prevalence to the included analytic sample, with some differences by race and ethnicity (eTable 1 in Supplement 1).

Among the included population, women were slightly older than men. The racial and ethnic composition was similar between sexes, with non-Hispanic White representing the majority in both groups. Notable sex differences were observed in behavioral risk factors; smoking was more prevalent among men (48.5% [95% CI, 45.8%-51.3%]) than women (36.2% [95% CI 33.6%-38.9%]), and a higher proportion of men exceeded the moderate alcohol use threshold compared with women (10.3% [95% CI, 8.6%-12.4%] vs 6.1% [95% CI, 4.9%-7.5%]). The prevalence of significant fibrosis was 6.9% (95% CI, 5.4%-8.8%) in women and 10.7% (95% CI, 8.8%-12.9%) in men (Table 1).

**Table 1.** Demographic and Cardiometabolic Characteristics by Sex

Characteristic	Men (n = 2989)		Women (n = 2992)	
	Unweighted No.	Weighted prevalence (95% CI), %	Unweighted No.	Weighted prevalence (95% CI), %
Age, mean (95% CI), y	2989	47 (46-48)	2992	49 (48-50)
Smoking	1488	48.5 (45.8-51.3)	995	36.2 (33.6-38.9)
Alcohol intake above moderate^a^	273	10.3 (8.6-12.4)	141	6.1 (4.9-7.5)
Race and ethnicity				
Hispanic	668	16.4 (13.2-20.2)	669	14.7 (12.0-18.0)
Non-Hispanic Black	728	9.4 (7.3-11.9)	769	11.0 (8.2-14.7)
Non-Hispanic White	1097	64.6 (59.6-69.3)	1073	65.0 (59.4-70.3)
Other^b^	496	9.6 (8.0-11.6)	481	9.2 (7.0-12.0)
Obesity	1243	42.3 (37.9-46.9)	1400	41.9 (38.8-45.0)
High WC^c^	1436	48.6 (44.3-53.1)	2146	69.0 (66.1-71.7)
Hypertension or antihypertensive medication	1600	44.9 (41.5-48.4)	1414	41.0 (38.2-43.8)
Glucose intolerance or diabetes medication	1299	41.7 (38.6-44.9)	1036	31.1 (28.7-33.5)
Hypertriglyceridemia or lipid-lowering agent	1511	48.8 (44.6-52.9)	1138	36.6 (34.0-39.3)
Low HDL level or lipid-lowering agent	1379	39.7 (35.9-43.6)	1283	42.6 (39.0-46.2)
≥2 CMRFs	2013	64.0 (60.2-67.7)	2017	63.6 (60.7-66.5)
Significant liver fibrosis (≥8 kPa)	343	10.7 (8.8-12.9)	243	6.9 (5.4-8.8)

Abbreviations: CMRF, cardiometabolic risk factor; HDL, high-density lipoprotein; WC, waist circumference.

^a^ Defined as greater than 20 g/d for women and greater than 30 g/d for men.

^b^ Other race indicates non-Hispanic other races, including Asian and multiple races.

^c^ Greater than 102 cm in men or 88 cm in women.

### Prevalence of CMRFs Among Women vs Men

Sex-based differences were observed in the prevalence of CMRFs (Table 1). While the overall prevalence of obesity was similar between women and men (41.9% [95% CI, 38.8%-45.0%] vs 42.3% [95% CI, 37.9%-46.9%]), women exhibited a higher prevalence of high WC (69.0% [95% CI, 66.1%-71.7%] vs 48.6% [95% CI, 44.3%-53.1%]). Conversely, men had higher prevalence of glucose intolerance (41.7% [95% CI, 38.6%-44.9%] vs 31.1% [95% CI, 28.7%-33.5%]), hypertension (44.9% [95% CI, 41.5%-48.4%] vs 41.0% [95% CI, 38.2%-43.8%]), and hypertriglyceridemia (48.8% [95% CI, 44.6%-52.9%] vs 36.6% [95% CI, 34.0%-39.3%]). The presence of low HDL cholesterol levels (42.6% [95% CI, 39.0%-46.2%] vs 39.7% [95% CI, 35.9%-43.6%]) and 2 or more CMRFs (63.6% [95% CI, 60.7%-66.5%] vs 64.0% [95% CI, 60.2%-67.7%]) were similar for women and men.

### Prevalence of Significant Fibrosis by CMRF

The sex-specific prevalence of significant fibrosis was generally lower for women compared with men across all CMRFs (Table 2). Most notably, among participants with high WC, women had a significant fibrosis prevalence of 9.7% (95% CI, 7.6%-12.5%) while men exhibited a higher prevalence of 17.4% (95% CI, 13.6%-21.9%; *P* = .001). A similar pattern of significant fibrosis prevalence was observed among women vs men for hypertriglyceridemia (9.9% [95% CI, 7.6%-13.0%] vs 13.6% [95% CI, 10.9%-16.9%]; *P* = .048) and low HDL cholesterol levels (9.8% [95% CI, 7.3%-13.0%] vs 13.6% [95% CI, 10.5%-17.5%]; *P* = .03). Alternatively, fibrosis prevalence was similar by sex among individuals with hypertension and glucose intolerance.

**Table 2.** Prevalence of Significant Liver Fibrosis by CMRF and Sex

CMRF	Men (n = 2989)		Women (n = 2992)		*P* value
	Unweighted, No.	Weighted prevalence of fibrosis (95% CI), %	Unweighted, No.	Weighted prevalence of fibrosis (95% CI), %	
Obesity	232	19.0 (14.4-24.7)	196	14.0 (10.4-18.5)	.10
High WC^a^	256	17.4 (13.6-21.9)	229	9.7 (7.6-12.5)	.001
Hypertension or antihypertensive medication	242	14.6 (12.3-17.3)	144	12.2 (9.4-15.8)	.26
Glucose intolerance or diabetes medication	197	13.6 (10.6-17.3)	135	12.9 (9.3-17.7)	.76
Hypertriglyceridemia or lipid-lowering agent	216	13.6 (10.9-16.9)	130	9.9 (7.6-13.0)	.048
Low HDL level or lipid-lowering agent	187	13.6 (10.5-17.5)	161	9.8 (7.3-13.0)	.03
≥2 CMRFs	296	13.9 (11.3-17.0)	226	10.3 (8.0-13.3)	.06

Abbreviations: CMRF, cardiometabolic risk factor; HDL, high-density lipoprotein; WC, waist circumference.

^a^ Greater than 102 cm in men or 88 cm in women.

### Association of CMRFs With Significant Fibrosis Among Women vs Men

**Figure 2.**
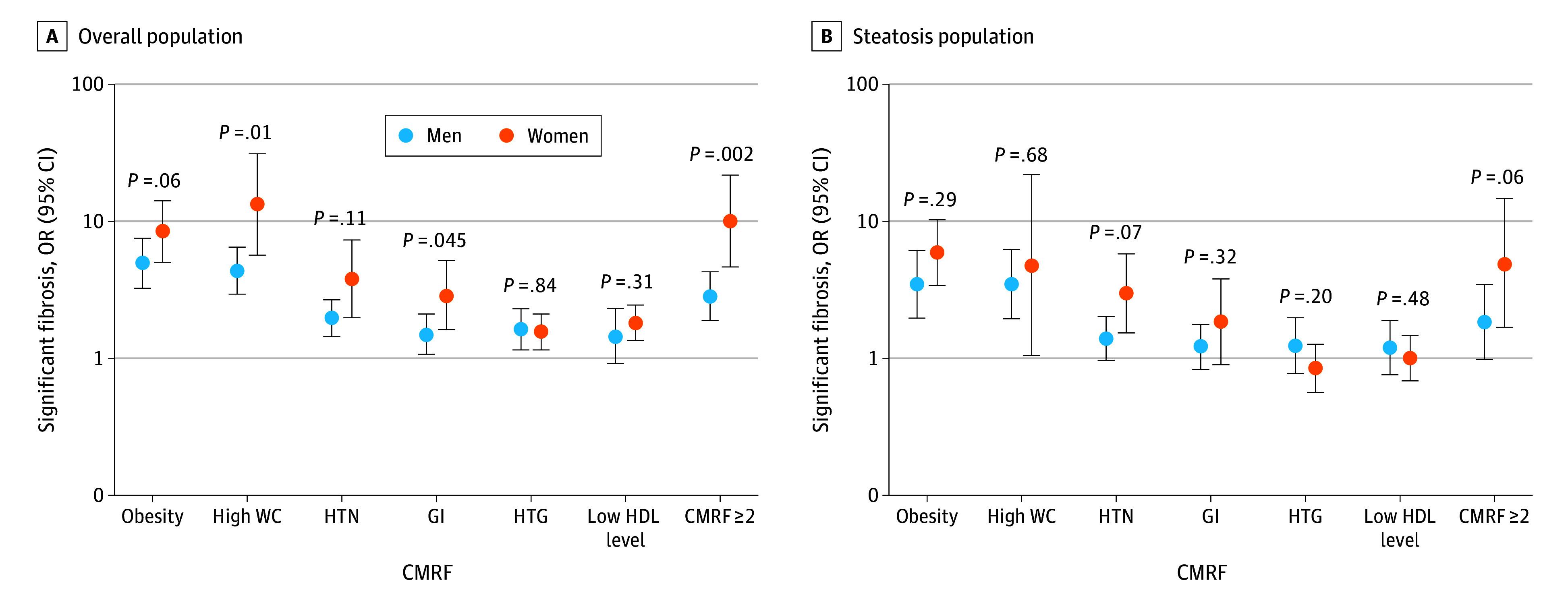
Dot Plot for Odds of Significant Liver Fibrosis by Sex Associated With Cardiometabolic Risk Factors (CMRFs) Odds are shown by adjusted odds ratios (aORs) in overall (A) and steatosis (B) populations. GI indicates glucose intolerance; HDL, high-density lipoprotein; HTG, hypertriglyceridemia, HTN, hypertension; WC, waist circumference.

While the estimated probability of significant fibrosis was generally higher for participants with vs without each CMRF among both sexes, women compared with men had higher odds of clinically significant fibrosis in association with specific CMRFs (Table 3). This was true for high WC (13.45 [95% CI, 5.70-31.78] vs 4.44 [95% CI, 3.00-6.57]; *P* for interaction = .01), glucose intolerance (2.94 [95% CI, 1.64-5.28] vs 1.51 [95% CI, 1.08-2.13]; *P* for interaction = .045), and presence of 2 or more CMRFs (10.22 [95% CI, 4.76-21.95] vs 2.87 [95% CI, 1.91-4.31]; *P* for interaction = .002) (Figure 2A). In contrast, point estimates for the association with significant fibrosis did not differ significantly in women vs men for obesity, hypertension, hypertriglyceridemia, and low HDL levels (Table 3). Results were similar in a secondary analysis adjusting for poverty (eTable 2 in Supplement 1).

**Table 3.** Multivariable Adjusted Odds and Age-Standardized Prevalence of Significant Liver Fibrosis

CMRFs	Men (n = 2989)				Women (n = 2992)				*P* for interaction^c^
	Age-standardized fibrosis prevalence (95% CI), %		aOR (95% CI)^b^	*P* value	Age-standardized fibrosis prevalence (95% CI), %		aOR (95% CI)^b^	*P* value	
	CMRF^a^	No CMRF^a^			CMRF^a^	No CMRF^a^			
Obesity	18.8 (14.2-24.5)	4.4 (3.3-5.9)	5.00 (3.28-7.62)	<.001	13.5 (10.2-17.7)	1.6 (1.1-2.2)	8.55 (5.13-14.26)	<.001	.06
High WC^d^	17.0 (13.3-21.4)	4.4 (3.2-6.1)	4.44 (3.00-6.57)	<.001	9.2 (7.2-11.8)	0.8 (0.4-1.6)	13.45 (5.70-31.78)	<.001	.01
Hypertension or antihypertensive medication	13.7 (11.1-16.7)	7.7 (5.6-10.6)	1.99 (1.47-2.71)	<.001	13.7 (10.4-18.0)	3.5 (2.3-5.4)	3.84 (1.98-7.44)	<.001	.11
Glucose intolerance or diabetes medication	11.9 (8.9-15.8)	8.7 (6.8-11.0)	1.51 (1.08-2.13)	.02	11.8 (7.8-17.4)	4.2 (2.8-6.2)	2.94 (1.64-5.28)	.001	.045
High triglyceride level or lipid-lowering agent	13.2 (10.5-16.5)	8.3 (6.3-10.8)	1.65 (1.17-2.33)	.006	9.4 (6.7-12.9)	5.3 (4.0-7.0)	1.58 (1.17-2.14)	.004	.84
Low HDL level or lipid-lowering agent	12.9 (9.8-16.6)	9.0 (6.7-12.0)	1.47 (0.93-2.34)	.10	9.4 (6.8-13.0)	4.8 (3.7-6.1)	1.84 (1.36-2.48)	<.001	.31
CMRF ≥2 vs 0-1	13.3 (10.8-16.4)	5.1 (3.4-7.5)	2.87 (1.91-4.31)	<.001	10.1 (7.7-13.1)	1.2 (0.6-2.3)	10.22 (4.76-21.95)	<.001	.002

Abbreviations: aOR, adjusted odds ratio; CMRF, cardiometabolic risk factor; HDL, high-density lipoprotein; WC, waist circumference.

^a^ *CMRF* and *No CMRF* refer to the presence or absence, respectively, of the CMRF listed in each row.

^b^ Multivariable models include a single CMRF adjusted for age, sex, race and ethnicity, smoking, and volume of alcohol use with sex interaction terms by each covariate to generate sex-specific aORs from a single pooled model.

^c^ *P* value for sex by cardiometabolic factor interaction.

^d^ Greater than 102 cm in men or 88 cm in women.

### Sensitivity Analyses

In the sensitivity analysis among 2452 participants with severe steatosis (Figure 2B), results were similar to the main analysis, although attenuated. The aOR for significant fibrosis was greater in women than in men in the presence of 2 CMRFs, although this difference was not statistically significant (4.88 [95% CI, 1.62-14.74] vs 1.83 [95% CI, 0.98-3.42]; *P* for interaction = .07) (eTable 3 in Supplement 1).

In the second sensitivity analysis, excluding participants with excessive alcohol use, results were similar to the main analysis. There were statistically significant differences between women and men in the aOR for high WC (13.22 [95% CI, 5.55-31.50] vs 4.58 [95% CI, 3.01-6.95]; *P* for interaction = .02) and the presence of 2 or more CMRFs (10.07 [95% CI, 4.70-21.60] vs 2.88 [95% CI, 1.88-4.40]; *P* for interaction = .003) in the association with significant fibrosis (eTable 4 in Supplement 1).

In a third sensitivity analysis, restricted to 2009 participants with MASLD, sex-specific associations between CMRFs and significant fibrosis were broadly consistent with primary findings, although estimates and differences by sex were attenuated. As observed in the main analysis, the aOR in the association with significant fibrosis was higher in women than men in the presence of 2 or more CMRFs (5.11 [95% CI, 1.28-20.36] vs 1.65 [95% CI, 0.69-3.96]), although there was no interaction between sex and 2 or more vs 0 to 1 CMRFs (*P* = .11). Additionally, hypertension showed an association with fibrosis with a significantly higher aOR in women than men (2.88 [95% CI, 1.53-5.42] vs 1.17 [95% CI, 0.71-1.92]; *P* for interaction = .03). Other CMRFs, including hypertriglyceridemia and low HDL cholesterol levels, were not associated with fibrosis in either sex within the MASLD subgroup, and aORs for some CMRFs, such as those for high WC and glucose intolerance, were nonsignificant among women (eTable 5 in the Supplement).

To further explore body roundness given the high WC findings, we calculated the BRI and examined its association with significant fibrosis as an alternative measure of central adiposity. We found that high BRI was common, observed in 73% (95% CI, 69.4%-76.6%) of men and 55% (95% CI, 51.6%-58.9%) of women. The prevalence of significant fibrosis among individuals with high BRI vs low BRI was similar in men (13.3% [95% CI, 10.6%-16.4%] vs 3.7% [95% CI, 2.4%-5.7%]) and women (11.8% [95% CI, 8.9%-15.3%] vs 1.0% [95% CI, 0.4%-2.1%]; *P* = .41). In multivariable logistic regression adjusting for age, race and ethnicity, smoking, and alcohol use, high BRI was associated with a substantial increase in risk of fibrosis in both sexes, with a higher aOR among women (12.4 [95% CI, 4.5-34.1]) compared with men (3.7 [95% CI, 2.1-6.4]; *P* for interaction = .006).

## Discussion

In this nationally representative US cross-sectional study, we identified key differences in CMRFs that are associated with significant liver fibrosis among women vs men. Women with 2 or more CMRFs had markedly higher odds of significant fibrosis compared with women with fewer risk factors (aOR, 10.22 [95% CI, 4.76-21.95]), whereas the corresponding association in men had a more modest aOR (2.87 [95% CI, 1.91-4.31]). Although these large aORs should be interpreted with caution given the cross-sectional context and overall low fibrosis prevalence, the magnitude of the differential association underscores the potential importance of sex as a determinant of metabolic risk stratification for liver disease. This aligns with a growing body of literature suggesting that women may have a lower threshold for metabolic injury in the context of clinically significant liver fibrosis, potentially due to a sexually dimorphic response to cardiometabolic stress.^2^ These findings suggest that in the presence of metabolic burden, women may experience a more pronounced fibrosis risk.

Notably, this pattern remained consistent in several sensitivity analyses, including among participants with severe steatosis and those without excessive alcohol use, reinforcing the robustness of the sex-specific associations. Among participants with MASLD, several individual CMRFs, particularly cholesterol-related factors, were no longer associated with fibrosis in either sex. This potentially reflects that once steatotic liver disease is established, fibrosis risk becomes less dependent on isolated metabolic traits. The directional pattern of increased risk among women with multiple CMRFs persisted for MASLD, consistent with our primary findings and underscoring that sex may modify metabolic susceptibility even within etiologically defined subgroups.^35,36^

Among individual CMRFs, central adiposity (measured by high WC) for women was associated with higher odds of significant fibrosis compared with women without elevated central adiposity (aOR, 13.45), triple the corresponding estimate in men (aOR, 4.44). This reflects age-standardized significant fibrosis prevalences of 9.2 (95% CI, 7.2-11.8) and 0.8 (95% CI, 0.4-1.6) for women with and without high WC, respectively, compared with 17.0 (95% CI, 13.3-21.4) and 4.4 (95% CI, 3.2-6.1) for men. Furthermore, women in our study had a markedly higher prevalence of high WC (69.0%) compared with men (48.6%), despite similar rates of overall obesity. Moreover, the association of overall obesity on fibrosis was not significantly different by sex. This highlights that central adiposity may be more critical than general obesity for liver fibrosis in women. This hypothesis was additionally supported by our assessment of BRI, which also demonstrated a greater increase in odds in the association with fibrosis among women. Interestingly, although high BRI was more prevalent in men, its association with fibrosis had a significantly higher aOR in women, suggesting that the adverse association of visceral adiposity per unit of body roundness may be heightened in women. This aligns with evidence that sex differences in visceral adipose tissue biology, such as its inflammatory potential and association with hepatic insulin resistance, may render women more susceptible to fibrosis at lower absolute levels of adiposity.^37,38,39^ One plausible explanation is the differential distribution and metabolic activity of visceral adipose tissue vs subcutaneous adipose tissue. Greater visceral adiposity, even in individuals with normal BMI, is strongly associated with hepatic insulin resistance, dyslipidemia, lipotoxicity, and elevated proinflammatory cytokine secretion, all of which contribute to liver fibrogenesis.^10,40,41^ Women generally have more subcutaneous adipose tissue than men, but this advantage diminishes after menopause, when estrogen levels decline and visceral adipose tissue accumulation increases.^2^ Estrogen is thought to exert hepatoprotective effects by modulating inflammation, insulin sensitivity, and fibrogenic pathways.^42,43^ Loss of estrogen with menopause leads to reduced adiponectin levels and increased inflammatory cytokine production, which may promote fibrotic remodeling in the liver.^44^ Additionally, sex-specific differences in insulin signaling and lipid metabolism may further exacerbate hepatic vulnerability to metabolic stress in women.^45,46^ These potential mechanisms offer biologic plausibility for our observation that high WC was associated with a significantly greater increase in odds of significant fibrosis in women than in men.

From a clinical perspective, this supports expanding beyond approaches that focus on BMI, which did not achieve statistical significance when comparing the association by sex in this study. This is particularly true for women, who may not meet traditional obesity thresholds but are metabolically at high risk. Emerging evidence highlights the importance of body shape and visceral adiposity, as measured by metrics like the BRI or imaging-based assessments of visceral fat, in identifying metabolic and inflammatory derangements independent of BMI.^47,48,49^ These findings underscore the need to integrate anthropometric and imaging tools that better capture visceral fat distribution and its metabolic consequences, particularly in women who exhibit high-risk adiposity phenotypes despite lower BMI.^37,50,51^

Beyond central adiposity, our analysis revealed glucose intolerance to have a larger aOR in the association with significant liver fibrosis in women than in men. This finding aligns with emerging evidence of sex-specific differences in insulin sensitivity and glucose metabolism.^44,46^ Women generally exhibit greater insulin sensitivity than men; however, when glucose intolerance develops, the relative loss of this metabolic advantage may render women at greater risk of hepatic injury.^52^ In states of glucose dysregulation, women demonstrate increased hepatic de novo lipogenesis, oxidative stress, and proinflammatory cytokine release compared with men, and all of these factors may worsen hepatic fibrosis.^44^ Additionally, impaired insulin signaling in women has been shown to drive greater visceral-to-subcutaneous fat redistribution, amplifying liver exposure to free fatty acids and inflammatory mediators.^50^ Our data specifically demonstrate that even with comparable levels of glucose intolerance, women face a disproportionately higher risk of significant liver fibrosis.

Perhaps the most compelling finding of our study was the markedly higher increase in the likelihood of significant liver fibrosis in women vs men with the presence of 2 or more CMRFs, with an aOR more than 3-fold higher in women than in men. This finding reinforces the recommendations of major guidelines regarding the need for fibrosis risk assessment in individuals with 2 or more metabolic risk factors.^17^ While underlying mechanisms contributing to this sex difference will require additional study, it is likely that the complex interplay of hormonal, genetic, and environmental factors collectively exacerbates to liver injury and fibrosis.^11^ The clinical implication is important; women and physicians who care for them need to be aware of the observed association between increasing numbers of CMRFs and higher odds of significant liver fibrosis. These findings highlight a potential need for future research to evaluate whether sex-specific risk stratification models could improve early detection. Our findings may be relevant for the early detection and management of liver fibrosis. Given the disproportionate association of central adiposity and glucose intolerance, further investigation is warranted to determine whether sex-specific considerations may be relevant for future risk stratification and clinical decision-making. This could involve evaluating whether lower thresholds for noninvasive fibrosis assessment in women with central adiposity or glucose intolerance are associated with improved risk stratification. For instance, while BMI is a common metric, our data, particularly the large aOR in the association of high WC with fibrosis, suggest that measures of central adiposity may be more informative for women. Our findings suggest that a one-size-fits-all approach to metabolic risk assessment in liver health may not fully capture sex-related differences, highlighting the potential value of further evaluating sex-specific approaches for effective prevention and intervention. The use of a nationally representative population and standardized assessments of metabolic and liver fibrosis markers enhances the generalizability and validity of our findings.

### Limitations

Our study, while robust, has several limitations that warrant consideration. First, its cross-sectional design precludes the establishment of causal inferences regarding the association between CMRFs and significant liver fibrosis. Longitudinal studies are needed to elucidate the temporal association and progression of fibrosis by sex-specific metabolic risk factors. Second, fibrosis staging was based on noninvasive indices (transient elastography) rather than histopathology. While transient elastography is a widely validated tool for assessing liver stiffness, it may introduce misclassification. Third, due to limitations in sample size, we were unable to stratify women by menopausal status or hormone therapy use. Hormonal transitions, particularly the decline in estrogen after menopause, are known to significantly impact metabolic profiles and liver health, and future research with larger cohorts should explore these nuances. Fourth, other potential behavioral and clinical risk factors for liver disease, such as dietary intake, toxins, and medication use, were not assessed. However, we have captured the primary exposures (cardiometabolic exposures and alcohol) contributing to nonviral chronic liver disease. Fifth, no adjustments were made for multiple comparisons; therefore, findings with *P* values near .05 should be interpreted cautiously as hypothesis generating rather than definitive evidence of sex differences.

## Conclusions

In this cross-sectional study of US adults, we found that the presence of multiple CMRFs was associated with a greater increase in odds of significant liver fibrosis in women than in men; this was especially true of high WC and glucose intolerance. While these findings require confirmation in large prospective studies, this study highlights potential sex-specific differences that may inform future research on fibrosis risk assessment. Further work is needed to determine whether incorporating sex-specific considerations into metabolic risk evaluation or surveillance approaches is associated with improved early identification of advanced liver disease among women.^53,54,55^

## Notes

Supplement 1. eTable 1. Demographic Characteristics of Participants Included in the Analysis and Those Excluded Due to Missing Data, NHANES 2017-2020 eTable 2. Multivariable Adjusted Odds Ratios and Age-Standardized Prevalence of Significant Liver Fibrosis in NHANES Survey Years 2017-2020, Stratified by Sex, After Adjusting for Poverty (N = 5272) eTable 3. Multivariable Adjusted Odds Ratios and Age-Standardized Prevalence of Significant Liver Fibrosis in NHANES Survey Years 2017-2020, Stratified by Sex, Among the Subgroup With Severe Steatosis (N = 2452) eTable 4. Multivariable Adjusted Odds Ratios and Age-Standardized Prevalence of Significant Liver Fibrosis in NHANES Survey Years 2017-2020, Stratified by Sex, Among the Subgroup Without Excessive Alcohol Use (N = 5865) eTable 5. Multivariable Adjusted Odds Ratios and Age-Standardized Prevalence of Significant Liver Fibrosis in NHANES Survey Years 2017-2020, Stratified by Sex, Among the Subgroup with MASLD (N = 2009)

Supplement 2.
Data Sharing Statement
