# Supplementary material for: Sex-Specific Cardiometabolic Profiles and Severity of Liver Fibrosis
Source: JAMA Netw Open. 2026 Mar 9;9(3):e260863. doi: 10.1001/jamanetworkopen.2026.0863 (PMC12973100; doi:10.1001/jamanetworkopen.2026.0863)
Supplement: Supplement 1. — eTable 1. Demographic Characteristics of Participants Included in the Analysis and Those Excluded Due to Missing Data, NHANES 2017-2020 eTable 2. Multivariable Adjusted Odds Ratios and Age-Standardized Prevalence of Significant Liver Fibrosis in NHANES Survey Years 2017-2020, Stratified by Sex, After Adjusting for Poverty (N = 5272) eTable 3. Multivariable Adjusted Odds Ratios and Age-Standardized Prevalence of Significant Liver Fibrosis in NHANES Survey Years 2017-2020, Stratified by Sex, Among the Subgroup With Severe Steatosis (N = 2452) eTable 4. Multivariable Adjusted Odds Ratios and Age-Standardized Prevalence of Significant Liver Fibrosis in NHANES Survey Years 2017-2020, Stratified by Sex, Among the Subgroup Without Excessive Alcohol Use (N = 5865) eTable 5. Multivariable Adjusted Odds Ratios and Age-Standardized Prevalence of Significant Liver Fibrosis in NHANES Survey Years 2017-2020, Stratified by Sex, Among the Subgroup with MASLD (N = 2009) [file jamanetwopen-e260863-s001.pdf]

Supplemental Online Content

Albhaisi S, Kim S, Terrault N, Dodge JL. Sex-specific cardiometabolic profiles and severity of liver fibrosis. *JAMA Netw Open*. 2026;9(3):e260863. doi:10.1001/jamanetworkopen.2026.0863

eTable 1. Demographic Characteristics of Participants Included in the Analysis and Those Excluded Due to Missing Data, NHANES 2017-2020

eTable 2. Multivariable Adjusted Odds Ratios and Age-Standardized Prevalence of Significant Liver Fibrosis in NHANES Survey Years 2017-2020, Stratified by Sex, After Adjusting for Poverty (N = 5272)

eTable 3. Multivariable Adjusted Odds Ratios and Age-Standardized Prevalence of Significant Liver Fibrosis in NHANES Survey Years 2017-2020, Stratified by Sex, Among the Subgroup With Severe Steatosis (N = 2452)

eTable 4. Multivariable Adjusted Odds Ratios and Age-Standardized Prevalence of Significant Liver Fibrosis in NHANES Survey Years 2017-2020, Stratified by Sex, Among the Subgroup Without Excessive Alcohol Use (N = 5865)

eTable 5. Multivariable Adjusted Odds Ratios and Age-Standardized Prevalence of Significant Liver Fibrosis in NHANES Survey Years 2017-2020, Stratified by Sex, Among the Subgroup with MASLD (N = 2009)

This supplemental material has been provided by the authors to give readers additional information about their work.

© 2026 Albhaisi S et al. *JAMA Network Open*.

eTable 1. Demographic Characteristics of Participants Included in the Analysis and those Excluded Due to Missing Data, NHANES 2017–2020.

**Characteristic**	**Included (N=5981)**	**Excluded due to missing data[a] (N=1295)**	***P*-value**
Age, mean (95% CI)[b]	48 (47-49)	47 (46-48)	.30
Female, % (95% CI)[b]	50.2 (48.2-52.2)	55.5 (50.8-60.1)	.05
Race/Ethnicity, % (95% CI)[b]			<.001
Non-Hispanic White	64.8 (59.7-69.6)	49.7 (42.5-56.8)	
Non-Hispanic Black	10.2 (7.8-13.2)	17.2 (12.6-22.9)	
Hispanic	15.6 (12.7-18.9)	20.0 (15.7-25.1)	
Other[c]	9.4 (7.6-11.6)	13.2 (9.9-17.4)	
Poverty, % (95% CI)[b]	41.9 (39.4-44.5)	41.0 (35.8-46.5)	.69

[a] Excluded due to missing responses for alcohol use or cardiometabolic data.

[b] Weighted mean or prevalence with 95% confidence interval (CI).

[c] Other race indicates non-Hispanic other races, including Asian and multiple races.

© 2026 Albhaisi S et al. *JAMA Network Open*.

eTable 2. Multivariable Adjusted Odds Ratios and Age-Standardized Prevalence of Significant Liver Fibrosis in NHANES Survey Years 2017-2020, Stratified by Sex, After Adjusting for Poverty (N=5272).

	**Men (N=2614)**				**Women (N=2658)**				**Interaction**
**CMRFs**	**Age-Standardized Prevalence of Significant Fibrosis (95% CI), %**		**Odds Ratio[a] (95% CI)**	***P***	**Age-Standardized Prevalence of Significant Fibrosis (95% CI), %**		**Odds Ratio[a] (95% CI)**	***P***	***P*[b]**
	**CMRF[c]**	**No CMRF[c]**			**CMRF[c]**	**No CMRF[c]**			
Obesity	18.6 (14.0-24.5)	4.2 (3.4-5.2)	5.18 (3.40-7.90)	<.001	14.0 (10.4-18.4)	1.6 (1.1-2.3)	8.58 (5.00-14.71)	<.001	.08
High waist circumference	16.7 (13.1-21.0)	4.3 (3.3-5.7)	4.45 (3.07-6.45)	<.001	9.7 (7.5-12.5)	0.6 (0.3-1.2)	18.30 (7.68-43.58)	<.001	.002
Hypertension or anti-hypertensive meds	14.4 (11.6-17.7)	6.6 (4.7-9.1)	2.52 (1.81-3.53)	<.001	15.4 (12.0-19.6)	3.6 (2.3-5.7)	4.21 (2.02-8.78)	<.001	.26
Glucose intolerance/DM meds	11.6 (8.6-15.6)	8.5 (6.9-10.4)	1.52 (1.08-2.14)	.02	12.4 (8.1-18.3)	4.3 (2.8-6.5)	3.10 (1.69-5.68)	.001	.03
High triglycerides or lipid lowering agent	13.6 (10.9-16.8)	7.6 (5.7-9.9)	1.91 (1.32-2.77)	.001	10.0 (7.3-13.5)	5.5 (4.1-7.4)	1.62 (1.20-2.18)	.003	.49
Low HDL or lipid lowering agent	13.0 (10.0-16.8)	8.6 (6.4-11.5)	1.58 (0.98-2.55)	.06	9.8 (7.0-13.6)	5.0 (3.9-6.5)	1.81 (1.33-2.45)	<.001	.57
CMRF ≥2 vs 0-1	13.6 (11.0-16.6)	4.0 (2.8-5.7)	3.51 (2.37-5.19)	<.001	10.7 (8.2-13.9)	1.0 (0.6-1.7)	11.68 (6.19-22.04)	<.001	<.001

[a] Multivariable models include a single CMRF adjusted for age, sex, race and ethnicity, smoking, volume of alcohol use, and poverty with sex interaction terms by each covariate to generate sex-specific odds ratios from a single pooled model.

[b] *P*-value for sex by cardiometabolic factor interaction.

[c] *CMRF* and *No CMRF* refer to the presence or absence, respectively, of the CMRF listed in each row.

© 2026 Albhaisi S et al. *JAMA Network Open*.

eTable 3. Multivariable Adjusted Odds Ratios and Age-Standardized Prevalence of Significant Liver Fibrosis in NHANES Survey Years 2017-2020, Stratified by Sex, Among the Subgroup with Severe Steatosis (N=2452).

	Men (N=1376)				Women (N=1076)				Interaction
**CMRFs**	**Age-Standardized Prevalence of Significant Fibrosis (95% CI), %**		**Odds Ratio (95% CI)**	***P***	**Age-Standardized Prevalence of Significant Fibrosis (95% CI), %**		**Odds Ratio (95% CI)**	***P***	***P*[b]**
	**CMRF[c]**	**No CMRF[c]**			**CMRF[c]**	**No CMRF[c]**			
Obesity	23.5 (18.2-29.9)	7.5 (4.7-11.7)	3.47 (1.96-6.13)	<.001	19.9 (14.8-26.1)	2.8 (1.6-4.7)	5.92 (3.38-10.36)	<.001	.29
High waist circumference	22.5 (17.6-28.3)	7.0 (4.4-10.9)	3.48 (1.95-6.21)	<.001	16.2 (12.0-21.5)	2.8 (0.8-8.9)	4.77 (1.04-21.80)	.04	.68
Hypertension or anti-hypertensive meds	20.8 (16.7-25.5)	16.4 (11.5-22.8)	1.39 (0.96-2.01)	.08	26.9 (20.2-34.8)	8.9 (5.8-13.4)	2.98 (1.53-5.81)	.002	.07
Glucose intolerance/DM meds	18.2 (13.2-24.4)	17.4 (13.5-22.1)	1.21 (0.83-1.76)	.31	19.7 (12.2-30.2)	11.9 (7.6-18.1)	1.85 (0.90-3.78)	.09	.32
High triglycerides or lipid lowering agent	20.4 (16.2-25.5)	16.6 (11.6-23.1)	1.24 (0.77-1.99)	.36	14.6 (10.2-20.3)	15.7 (11.0-21.9)	0.84 (0.56-1.28)	.41	.20
Low HDL or lipid lowering agent	20.4 (16.3-25.4)	17.2 (12.3-23.6)	1.20 (0.76-1.89)	.41	16.0 (11.1-22.5)	14.4 (10.4-19.7)	1.00 (0.69-1.47)	.98	.48
CMRF ≥2 vs 0-1	20.2 (16.1-25.1)	13.4 (7.2-23.3)	1.83 (0.98-3.42)	.06	17.4 (13.0-22.9)	4.4 (1.6-11.1)	4.88 (1.61-14.74)	.007	.06

[a] Multivariable models include a single CMRF adjusted for age, sex, race and ethnicity, smoking, and volume of alcohol use with sex interaction terms by each covariate to generate sex-specific odds ratios from a single pooled model.

[b] *P*-value for the sex by cardiometabolic factor interaction.

[c] *CMRF* and *No CMRF* refer to the presence or absence, respectively, of the CMRF listed in each row.

© 2026 Albhaisi S et al. *JAMA Network Open*.

eTable 4. Multivariable Adjusted Odds Ratios and Age-Standardized Prevalence of Significant Liver Fibrosis in NHANES Survey Years 2017-2020, Stratified by Sex, Among the Subgroup Without Excessive Alcohol Use (N=5865).

	**Men (N=2894)**				**Women (N=2971)**				**Interaction**
**CMRFs**	**Age-Standardized Prevalence of Significant Fibrosis (95% CI), %**		**Odds Ratio (95% CI)**	***P***	**Age-Standardized Prevalence of Significant Fibrosis (95% CI), %**		**Odds Ratio (95% CI)**	***P***	***P*** [b]
	**CMRF**[c]	**No CMRF**[c]			**CMRF**[c]	**No CMRF**[c]			
Obesity	18.8 (14.0-24.8)	4.4 (3.2-5.9)	5.08 (3.22-8.03)	<.001	13.4 (10.1-17.5)	1.6 (1.2-2.2)	8.34 (5.03-13.84)	<.001	.09
High waist circumference	17.1 (13.3-21.6)	4.4 (3.1-6.1)	4.58 (3.01-6.95)	<.001	9.2 (7.2-11.7)	0.8 (0.4-1.6)	13.22 (5.55-31.50)	<.001	.02
Hypertension or anti-hypertensive meds	13.6 (11.0-16.7)	7.9 (5.6-10.9)	1.94 (1.42-2.65)	<.001	13.8 (10.5-17.9)	3.5 (2.3-5.4)	3.90 (1.97-7.74)	<.001	.08
Glucose intolerance/DM meds	12.0 (8.8-16.0)	8.5 (6.7-10.8)	1.54 (1.08-2.18)	.02	11.7 (7.8-17.3)	4.2 (2.8-6.2)	2.90 (1.60-5.24)	.001	.06
High triglycerides or lipid lowering agent	13.5 (10.6-17.1)	7.9 (6.0-10.4)	1.78 (1.21-2.60)	.005	9.3 (6.8-12.7)	5.3 (4.0-7.0)	1.58 (1.16-2.14)	.005	.62
Low HDL or lipid lowering agent	13.0 (10.0-16.9)	8.8 (6.5-11.9)	1.53 (0.96-2.44)	.07	9.3 (6.7-12.8)	4.8 (3.7-6.2)	1.79 (1.33-2.42)	<.001	.47
CMRF ≥2 vs 0-1	13.4 (10.8-16.5)	5.1 (3.4-7.6)	2.88 (01.88-4.40)	<.001	10.1 (7.7-13.0)	1.2 (0.6-2.3)	10.07 (4.70-21.60)	<.001	.003

[a] Multivariable models include a single CMRF adjusted for age, sex, race and ethnicity, and smoking with sex interaction terms by each covariate to generate sex-specific odds ratios from a single pooled model.

[b] *P*-value for the sex by cardiometabolic factor interaction.

[c] *CMRF* and *No CMRF* refer to the presence or absence, respectively, of the CMRF listed in each row.

© 2026 Albhaisi S et al. *JAMA Network Open*.

eTable 5. Multivariable Adjusted Odds Ratios and Age-Standardized Prevalence of Significant Liver Fibrosis in NHANES Survey Years 2017-2020, Stratified by Sex, Among the Subgroup with MASLD (N=2009).

	**Men (N=1117)**				**Women (N=892)**				**Interaction**
**CMRFs**	**Age-Standardized Prevalence of Significant Fibrosis (95% CI), %**		**Odds Ratio[a] (95% CI)**	***P***	**Age-Standardized Prevalence of Significant Fibrosis (95% CI), %**		**Odds Ratio[a] (95% CI)**	***P***	***P*[b]**
	**CMRF[c]**	**No CMRF[c]**			**CMRF[c]**	**No CMRF[c]**			
Obesity	24.3 (18.8-30.9)	7.0 (3.8-12.4)	3.58 (1.72-7.45)	.001	22.3 (16.6-29.2)	3.1 (1.7-5.9)	6.38 (3.46-11.75)	<.001	.32
High waist circumference	23.2 (18.1-29.3)	7.8 (4.1-14.3)	3.16 (1.46-6.86)	.005	18.6 (13.8-24.7)	3.8 (1.2-11.8)	4.55 (0.86-23.98)	.07	.68
Hypertension or anti-hypertensive meds	21.4 (16.9-26.8)	19.0 (12.8-27.2)	1.17 (0.71-1.92)	.52	29.2 (22.0-37.7)	10.3 (6.5-16.1)	2.88 (1.53-5.42)	.002	.03
Glucose intolerance/DM meds	19.6 (14.3-26.1)	18.0 (13.7-23.4)	1.30 (0.83-2.04)	.23	21.4 (13.5-32.4)	14.6 (9.6-21.8)	1.64 (0.85-3.19)	.14	.61
High triglycerides or lipid lowering agent	21.8 (16.6-28.2)	16.8 (10.7-25.5)	1.31 (0.68-2.53)	.40	16.6 (11.3-23.7)	19.4 (13.8-26.7)	0.75 (0.51-1.09)	.13	.10
Low HDL or lipid lowering agent	20.8 (16.4-26.1)	19.0 (12.9-27.2)	1.10 (0.64-1.86)	.72	17.6 (12.1-24.8)	18.7 (13.3-25.6)	0.88 (0.59-1.30)	.50	.45
CMRF ≥2 vs 0-1	21.1 (16.6-26.5)	15.7 (7.4-30.3)	1.65 (0.69-3.96)	.25	20.0 (15.0-26.2)	5.4 (1.6-16.6)	5.11 (1.28-20.36)	.02	.11

[a] Multivariable models include a single CMRF adjusted for age, sex, race and ethnicity, smoking, and volume of alcohol use with sex interaction terms by each covariate to generate sex-specific odds ratios from a single pooled model.

[b] *P*-value for the CMRF by sex interaction.

[c] *CMRF* and *No CMRF* refer to the presence or absence, respectively, of the CMRF listed in each row.

© 2026 Albhaisi S et al. *JAMA Network Open*.
