# Supplementary material for: Sex-Specific Cardiometabolic Profiles and Severity of Liver Fibrosis
Source: JAMA Netw Open. 2026 Mar 9;9(3):e260863. doi: 10.1001/jamanetworkopen.2026.0863 (PMC12973100; doi:10.1001/jamanetworkopen.2026.0863)
Supplement: Supplement 2. — Data Sharing Statement [file jamanetwopen-e260863-s002.pdf]

Data Sharing Statement

Albhaisi. Sex-Specific Cardiometabolic Profiles and Severity of Liver Fibrosis. *JAMA Netw Open*. Published online March 9, 2026. doi:10.1001/jamanetworkopen.2026.0863

Data
Data available: Yes
Data types: Deidentified participant data, Data dictionary
How to access data: Request for data to be sent to the study Principal Investigator (Jennifer Dodge, MPH) via Email: Jennifer.Dodge@med.usc.edu
When available: With publication

Supporting Documents
Document types: None

Additional Information
Who can access the data: Anyone requesting the data
Types of analyses: For any purpose
Mechanisms of data availability: With investigator support, after approval of a proposal
